# APOBEC3B, a molecular driver of mutagenesis in human cancers

**DOI:** 10.1186/s13578-017-0156-4

**Published:** 2017-05-30

**Authors:** Jun Zou, Chen Wang, Xiangyi Ma, Edward Wang, Guang Peng

**Affiliations:** 1 Department of Oncology, Tongji Hospital, Tongji Medical College, Huazhong University of Science and Technology, Wuhan, 430030 China; 2 Department of Obstetrics and Gynecology, Tongji Hospital, Tongji Medical College, Huazhong University of Science and Technology, Wuhan, 430030 China; 3 OncoMed Pharmaceuticals, 800 Chesapeake Dr., Redwood City, CA 94063 USA; 4 Department of Clinical Cancer Prevention, MD Anderson Cancer Center, The University of Texas, Houston, TX 77030 USA

**Keywords:** APOBEC, APOBEC3B, DNA editing, Mutagenesis, Human cancer

## Abstract

Human cancers results in large part from the accumulation of multiple mutations. The progression of premalignant cells is an evolutionary process in which mutations provide the fundamental driving force for genetic diversity. The increased mutation rate in premalignant cells allows selection for increased proliferation and survival and ultimately leads to invasion, metastasis, recurrence, and therapeutic resistance. Therefore, it is important to understand the molecular determinants of the mutational processes. Recent genome-wide sequencing data showed that apolipoprotein B mRNA editing catalytic polypeptide-like 3B (APOBEC3B) is a key molecular driver inducing mutations in multiple human cancers. APOBEC3B, a DNA cytosine deaminase, is overexpressed in a wide spectrum of human cancers. Its overexpression and aberrant activation lead to unexpected clusters of mutations in the majority of cancers. This phenomenon of clustered mutations, termed kataegis (from the Greek word for showers), forms unique mutation signatures. In this review, we will discuss the biological function of APOBEC3B, its tumorigenic role in promoting mutational processes in cancer development and the clinical potential to develop novel therapeutics by targeting APOBEC3B.

## Background

It is well known that the accumulation of diverse mutations is closely linked to the development of carcinogenesis [1, 2]. Cancer genomic sequencing studies have identified a variety of mutational signatures that reflect the corresponding causes of these mutations.

Mutagenesis originates from exogenous sources found in the environment, and endogenous sources that reside intracellularly [3, 4]. Exogenous sources include radiation and chemical damage. An example is cytosine to thymine (C-to-T) transitions caused by ultraviolet light and oxidative damage, which ultimately form pyrimidine dimers [5, 6]. Endogenous sources can be further divided into passive and active sources of DNA damage. Passive alteration is characterized by an inability to repair the DNA damage after it has been triggered. The active endogenous sources of mutation are agents that impair DNA directly, including hydrolytic deamination of cytosine [7].

Previous studies have shown that normal enzymatic activity in DNA repair systems can also be a major endogenous source of DNA injury and mutation in cancer, which adds to the complexity of the mechanisms of carcinogenesis [8]. Analyses of whole-genome and exome-wide mutation data files in The Cancer Genome Atlas (TCGA) have revealed that the existence of apolipoprotein B mRNA editing catalytic polypeptide-like (APOBEC) cytidine deaminase mutagenesis patterns could have a role in somatic mutations of carcinogenesis and ultimately lead to genome instability [9, 10].

## The biological function of the APOBEC family

A major contributor of mutations in many different tumor types is the APOBEC family of enzymatic DNA cytosine deaminases [11–14]. The APOBEC family came to light with the discovery that apolipoprotein B (apoB) mRNA included a cytosine to uracil (C-to-U) base modification that was not hereditarily encoded [15].

APOBEC family members normally functions as DNA mutators participating in the innate immune system that defends against their targets (retrovirus and retrotransposon) propagation. For instance, APOBEC proteins can inhibit human immunodeficiency virus type 1 (HIV-1) viral reverse transcription by DNA-editing dependent and independent processes [16–19]. The APOBEC family in most humans is composed of seven enzymes, each with conserved cytidine deaminase domains (CDAs). The human APOBEC family includes activation-induced cytosine deaminase (hAID), APOBEC1 (hA1), APOBEC2 (hA2), APOBEC3 (hA3A–hA3H) encoded in a tandem cluster on chromosome 22, and APOBEC4 on chromosome 1 [20, 21].

**Fig. 1:**
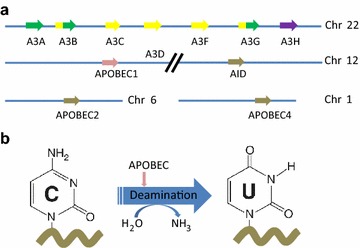
**a** The spatial location of genes encoding 11 members of APOBEC family in human. AID, APOBEC1, APOBEC3A, APOBEC3C, and APOBEC3H have single zinc-coordinating domains, whereas APOBEC3B, APOBEC3D, APOBEC3F, and APOBEC3G are double domain enzymes.* The colors* represent the different categories of catalytic domains in APOBECs. *Green* represents a Z1 catalytic domain, *yellow* represents a Z2 catalytic domain, and* violet* represents a Z3 catalytic domain. AID is represented by *pink*, and the rest is represented by *grey*. **b** APOBEC family enzymes catalyze the hydrolytic reaction of cytosine to uracil (C-to-U) in single-strand DNA (ssDNA) substrate

All enzymes of the AID/APOBEC family have at least one zinc-dependent catalytic domain, which contains the consensus amino acid sequence H-X-E-X23-28-P-C-X2-4-C (X stands for any amino acid) [22]. APOBEC3A, APOBEC3C, APOBEC3H, AID, and APOBEC1 have a single conserved zinc-dependent domain, while APOBEC3B, APOBEC3D, APOBEC3F, APOBEC3G have two conserved zinc-coordinating domains [14, 23] (Fig. 1a).

The intron/exon organization of the APOBEC3A to APOBEC3G genes was established by DNA sequencing and restriction enzyme mapping of the bacterial artificial chromosome (BAC) and P1-derived artificial chromosome (P1) clones. These genes include two eight-exon genes (APOBEC3B and 3G), one seven-exon gene (APOBEC3F), one five-exon gene (APOBEC3A), two four-exon genes (APOBEC3C and 3E), and one three-exon gene (APOBEC3D).

The primary biochemical reaction induced by APOBEC family proteins is cytosine to uracil (C-to-U) deamination (Fig. 1b). However, cytosine to guanine (C-to-G) transitions and other mutations can be induced by these enzymes [13, 14].

All of the APOBEC enzymes, except for APOBEC2 and APOBEC4, are capable of converting cytosine in ssDNA through a deamination reaction to uracil (C-to-U). The enzymatic deamination occurs at much faster rates on unprotected ssDNA substrates. However, different APOBEC enzymes with DNA editing activity can have independent physiological functions [24].

AID, emerging as one member of the first APOBECs, is a key enzyme in adaptive immunity for antibody diversity and affinity maturation. AID can initiate the somatic hypermutation and class-switch recombination of immunoglobulin genes. In addition, it can mutate chromosomal DNA at a limited number of secondary targets. This function of AID has been implicated in carcinogenesis [25, 26].

APOBEC1 is the first APOBEC family member to be identified and characterized as an RNA editor, which specifically deaminates mRNA in ApoB at cytosine6666 to uracil [27]. Other mRNA targets of APOBEC1 have been depicted recently, where the reciprocal action occurs at AU-rich sequence in 3′ untranslated regions (3′ UTRs) of diverse genes and modulates mRNA stability [28]. These physiological functions of APOBEC1 help explain mechanisms by which overexpression of APOBEC1 can initiate cancer [14].

APOBEC2 expression is well defined in the heart, skeletal muscle and tumor necrosis factor alpha (TNF-α) activated liver cells, however its precise physiological activity has yet to be determined [29–31]. As for APOBEC4, early and recent research has suggested that it may have a natural role in regulating host promoters or endogenous long terminal repeat (LTR) promoters [32].

The family members of genes encoding APOBEC3 proteins is positioned within a 200 kb APOBEC3 genomic cluster on human chromosome 22q13.1, and the corresponding protein function is to protect human cells against retroviruses and endogenous mobile retroelements as potent mutators of viral DNA [33]. Whereas the fundamental function of AID is in adaptive immunity, APOBEC3 members play an important part in innate immunity. Thus, APOBEC3 proteins are powerful forces against both endogenous and exogenous viruses. Nonetheless, they are closely involved in immunity in multiple ways. For example, DNA editing can be induced by A3G in adaptive immunity. Previous study designed to identify a host cell suppressor of the HIV-1 accessory protein, viral infectivity factor (VIF), reported its function as an antiviral host factor [19, 34]. A3G has also been shown to promote CD8+ cytotoxic T lymphocytes (CTL) recognition of infected T lymphatic cells and restrict marginal zone B cells, possibly resulting in a shift from a prompt immune response to a much more sustained germinal center B cell response [35]. Recent studies have shown that A3A induced by inflammation-related factors edits the mRNAs of thousands of genes, some associated with viral pathogenesis in macrophages and monocytes [36, 37]. Besides editing nuclear DNA or mitochondrial DNA and some transfected plasmids, A3A can also be involved in a novel G-to-A form of mRNA editing [38, 39].

## The biological function of APOBEC3B

In general, all APOBEC3 family members can lead to hypermutation of viral genomes, which are replicated via syntheses of ssDNA intermediates. The intron/exon boundaries of APOBEC3B, APOBEC3G, and APOBEC3F are in identical positions, except APOBEC3F terminates after exon 7. In APOBEC3B, APOBEC3G, and APOBEC3F exons 2, 3, and 4 are duplicated in exons 5, 6, and 7, so that introns 1–4 are in the same position as introns 5–7 [40].

On the basis of their structure, the APOBEC3 proteins are divided into two groups. APOBEC3B, APOBEC3D, APOBEC3F, and APOBEC3G contain two zinc-dependent cytidine deaminase domains (ZD-CDAs), instead of one in APOBEC3A, APOBEC3C, and APOBEC3H [23]. Although these deaminase domains are usually conserved, they can function and evolve independently. Thus these variations can promote evolutionary flexibility [23] (Fig. 1a).

It’s well known that APOBEC3B plays a crucial role in retrovirus and endogenous retrotransposon restriction by hyperediting complementary DNA (cDNA) intermediates [41]. A3B contains two CDAs, and there are controversial reports about whether both domains are required for full editing activity in restricting HIV-1, whereas only carboxyl-terminal CDA is required for blocking HBV replication and editing bacterial DNA [2, 42]. A recent study has demonstrated that only the carboxyl-terminal CDA has C deamination activity, and N-terminal CDA is inactive [43].

Since the discovery of the APOBEC DNA mutating features in 2002, the APOBEC proteins have been linked to cancer [17]. APOBEC3 cytidine deaminase activity has been proved being involved with tumor evolution and metastasis [44, 45]. Research has shown that three human APOBEC3 members are strictly cytoplasmic (APOBEC3D, APOBEC3F and APOBEC3G) because of selection for paralogs. Previous researches have shown that APOBEC3A, APOBEC3C and APOBEC3H exhibit both cytoplasmic and nuclear localizations, but APOBEC3B is expressed almost exclusively in the nucleus. APOBEC3A and APOBEC3B can deaminate nuclear DNA as well as 5-methyl-deoxycytidine (5-MeC) residues in ssDNA, with APOBEC3A being the more efficient [2, 43, 46–51]. Furthermore, AID and APOBEC3H also have been shown to deaminate MeC [52–56]. It has been reported that nuclear DNA editing caused by APOBEC3A up-regulation can lead to double stranded DNA (dsDNA) breaks and apoptosis [57, 58].

## The increased expression of APOBEC3B in human cancers

Increasing evidence have shown that APOBEC3B may be a predominant mutagenic agent having effects on the genesis and evolution of various cancers [4, 8, 48]. This DNA mutator hypothesis is supported by studies indicating that APOBEC3B expression is elevated in diverse forms of cancer tissues and cell lines [40, 48, 59], in contrast to its comparatively low levels in the corresponding normal human tissues spanning all major organs [8, 48, 59]. This hypothesis is also supported by its unique localization to nucleus, which can serve as a unique driving force for mutagenesis promoting tumor development [48, 60].

An in-depth analysis has shown that the APOBEC3B mutation signature is specifically enriched in at least six types of cancers, including those of the cervix, breast, lung (adeno and squamous cell), head and neck, and bladder [8, 61].

Recent observations linked DNA cytosine deaminase APOBEC3B to the mutational process driving breast carcinogenesis. These studies have demonstrated that APOBEC3B is a biomarker of poor prognosis and poor outcomes for estrogen receptor (ER)+  breast cancer, strongly indicating that genetic aberrations induced by APOBEC3B contribute to breast cancer progression [62–64]. Genetic, cellular and biochemical studies have demonstrated that APOBEC3B-catalyzed genomic uracil lesions are responsible for a large proportion of both dispersed and clustered mutations in multiple distinct cancers [8, 48, 61, 63, 65–79].

The observations of APOBEC3B overexpression in different forms of cancers are shown in Table 1.

**Table 1:** Overexpression of APOBEC3B in cancers

Cancer type	Discovery	Model	References
Breast cancer	Expression of APOBEC3B is increased in breast tumors and cell lines. Breast TCGA tumors have a more prevalent APOBEC3B mutation than is expected	Human tissue samples. In vitro, human cell lines	[8, 48, 64]
	HER2-enriched subtype of breast cancer has a significantly higher frequency of mutations associated with APOBEC3B than other breast cancer subtypes	TCGA	[60]
	APOBEC3B leads to drug resistance in breast cancer and APOBEC3B-dependent tumor evolvability may serve as a effective target to improve efficacies of anti-cancer therapies	Human tissue samples	[62, 65]
	APOBEC3B depletion in an ER+ breast cancer cell line results in prolonged tamoxifen response	Xenograft model	[66]
Gastric cancer	APOBEC3B expression was higher in gastric cancer tissues than that in normal tissues and APOBEC3B overexpression indicates the unfavorable prognosis of the patients with gastric cancer	Human tissue samples	[8, 62, 67]
Chondrosarcoma	APOBEC3B was overexpressed in chondrosarcoma tissues, and APOBEC3B deficiency caused slight apoptosis in the chondrosarcoma cells	Human tissue samples. In vitro, human cell lines	[8, 68]
Hepatocellular carcinoma	APOBEC3B was the only APOBEC3 family member significantly overexpressed in hepatocellular carcinoma (HCC) tissues and may be a potential factor contributing to suppression of tumor growth in HCC	Human tissue samples. In vitro, human cell lines	[69]
	APOBEC3B is a potential factor contributing to suppression of tumor growth in HCC	In vitro, human cell lines	[70]
Renal cancer	Renal clear-cell carcinomas showed statistically notable up-regulation of APOBEC3B	Human tissue samples	[8, 71]
Colorectal cancer	APOBEC3B was overexpressed in colorectal cancer tissues	Human tissue samples	[8, 72]
Prostate cancer	Prostate carcinomas showed statistically marked up-regulation of APOBEC3B	Human tissue samples	[8, 72]
Cervix cancer	APOBEC3B was overexpressed in cervix cancer tissues	Human tissue samples	[8]
Bladder cancer	APOBEC3B was overexpressed in bladder cancer tissues	Human tissue samples	[8, 73]
Lung cancer	The APOBEC3B expression is elevated obviously in non-small cell lung cancer (NSCLC) tissues and the overexpression of APOBEC3B was correlated with unfavorable prognosis	Human tissue samples	[8, 74]
	The tumor/normal ratio of APOBEC3B mRNA levels was not different within the sexuality, age, smoking status, epidermal growth factor receptor (EGFR), kirsten rat sarcoma viral oncogene (KRAS) mutation and pathological stages	Human tissue samples	[75]
Head and neck	The mRNA level of APOBEC3B were significantly higher in cancer tissues than in the corresponding noncancerous esophageal mucosae	Human tissue samples	[8, 76]
	APOBEC3B mRNA expression was significantly higher in oral squamous cell carcinomas (OSCC), compared to non-cancerous oral tissues	Human tissue samples	[77]
Ovarian cancer	APOBEC3B may paly a potential role in serous ovarian cancer genomic instability	Human tissue samples. In vitro, human cell lines	[78]

## The mutational process induced by APOBEC3B

**Fig. 2:**
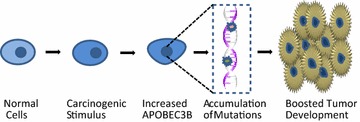
The simplified process of A3B-induced tumor development

Whether APOBEC3B mutagenic activity is a potential cancer driver or a downriver effector remains an open question, and the mechanism of APOBEC3B upregulation in cancer cells needs further evidence. The collective studies suggest that the up-regulation of APOBEC3B in developing tumors promotes cancer progression [12] (Fig. 2).

Many studies have demonstrated a positive correlation between a defined mutation signature and overexpression of APOBEC3B in many tumor types [8, 46, 53, 55, 57–71]. Furthermore, the cancer types expressing the highest levels of APOBEC3B are likely to have the highest frequency of mutations. It is essential for us to obtain a general understanding of the main mutations resulting from APOBEC3B cytosine deamination to uracil.

Based on the previous studies on AID, it is established that U:G mispairs resulting from cytosine deamination can result in all six base substitution mutations [80]. While many U:G lesions are likely repaired in an error-free manner by the canonical base excision repair pathway, lesions that escape this process have multiple distinct mutagenic potentials [81]. Simple DNA replication across uracilated DNA results in C-to-T transitions. Mutagenic mismatch repair (MMR) at U:G mispairs may result in transitions and/or transversions. Translesion DNA synthesis across abasic sites can result in transition mutations. The repair process may generate nicks on both strands of the DNA double helix that are relatively close to one another potentially resulting in double-stranded breaks.

In breast cancer, APOBEC3B upregulation correlated with increased levels of transition mutations, suggesting that a proportion of the genomic uracils created by APOBCE3B either persist through DNA synthesis or are generated at a high enough rate that they are detectable in non-replicated DNA [48]. If a uracil is not excised by a DNA glycosylase prior to DNA replication, it will template as a thymine and base pair with adenosine. After a subsequent round of DNA replication, the result is a C-to-T transition mutation.

C → T transitions in multiple human cancers have been suggested to be caused by APOBEC3B. A uracil residue results from APOBEC3B cytosine deamination can be excised by uracil DNA glycosylase and then generates an abasic site (AP site) leading to insertion of adenine opposite the AP site [82]. Thus APOBEC3B editing results in C → T transitions in carcinogenesis. Other processes like spontaneous or chemical-induced cytosine deamination, error-prone bypass can also create AP site and C → T transitions. APOBEC3B preferentially deaminates cytosine residues when it is adjacent to a 5′ thymine and a 3′ thymine or adenine [83]. Current studies have shown that only cytosine substitutions that occur within the trinucleotide TCA or TCT sequence context are attributed to APOBEC3B mutagenesis [4].

In addition to C deamination of APOBEC3B contributes to mutagenesis, recently studies have shown that a methionine residue at the joint of the carboxyl-terminal CDA and the N-terminal CDA has been proved to play a role in high mutagenicity [51, 84]. It has been established that the A3B’s capability of 5-MeC deamination is much less efficient than that of APOBEC3A [2, 43, 46–51]. Although the carboxyl-terminal CDA of APOBEC3B have been shown to comparatively weakly convert some 5-MeC into T in ssDNA substrates, the C-to-U deamination of APOBEC3B is much more efficient than that of APOBEC3A [51]. Multiple factors contributing to the 5-MeC deamination activity and specificity by APOBEC3B may promote mutagenesis [43, 51].

Studies have shown that a significantly large subset of Asian (37%), Amerindian (58%), and Oceania (93%) populations have a deletion in the APOBEC3B gene, which is associated with an approximate 20-fold increase in the expression of an APOBEC3A from an mRNA variant containing the 3′-UTR of APOBEC3B [85]. This 29.5 kB deletion between exon 5 in APOBEC3A and exon 8 in APOBEC3B is linked to increased risk for breast cancer, hepatocellular carcinoma (HCC) and epithelial ovarian cancer, whereas this deletion polymorphism is not involved with clinical outcome of mammary cancer regardless of APOBEC3B mRNA levels [13, 86–89].

## Conclusion

Above all, APOBEC3B may represent an important marker for various human cancers and a strong candidate for targeted intervention, especially given its essential nature to tumor progression and heterogeneity. Therefore APOBEC3B inhibition may decrease the rate of cancer progression and keep the stability of the targeted genome [48]. Future in-depth research is demanded to understand APOBEC3B protein regulation and the potential interaction with many other oncogenes and tumor suppressors. All studies of APOBEC3B in the last decade show that APOBEC3B will be a promising target for cancer prevention and therapy.

## Abbreviations

C-to-T: cytosine to thymine
TCGA: The Cancer Genome Atlas
APOBEC: apolipoprotein B mRNA editing catalytic polypeptide-like
AID: activation-induced cytidine deaminase
C-to-U: cytosine to uracil
HIV-1: human immunodeficiency virus type 1
CDAs: cytidine deaminase domains
C-to-G: cytosine to guanine
ssDNA: single-strand DNA
BAC: bacterial artificial chromosome
P1: P1-derived artificial chromosome
3′ UTRs: 3′ untranslated regions
TNF-α: tumor necrosis factor alpha
LTR: long terminal repeat
VIF: viral infectivity factor
CTL: cytotoxic T lymphocytes
G-to-A: guanine to adenine
ZD-CDAs: zinc-dependent cytidine deaminase domains
dsDNA: double stranded DNA
ER: estrogen receptor
HCC: hepatocellular carcinoma
NSCLC: non-small cell lung cancer
EGFR: epidermal growth factor receptor
KRAS: kirsten rat sarcoma viral oncogene
OSCC: oral squamous cell carcinomas
MMR: mismatch repair
AP site: abasic site
5-MeC: 5-methyl-deoxycytidine
